# Deciphering Early-Stage Molecular Mechanisms of Negative Pressure Wound Therapy in a Murine Model

**DOI:** 10.3390/ijms25042373

**Published:** 2024-02-17

**Authors:** Yu-Chiau Shyu, Ting-Shuo Huang, Hua-Sheng Chiu, Pavel Sumazin, Xin-Yu Lin, Po-Cheng Liao, Cai-Cin Liou, Fang-Chia Hsu, Jyuan-Siou Lin, Chih-Chin Hsu, Pang-Hung Hsu, Chi-Chin Sun, Chien-Tzung Chen

**Affiliations:** 1 Community Medicine Research Center, Chang Gung Memorial Hospital, Keelung Branch, Keelung 204, Taiwan; love20070810@gmail.com (X.-Y.L.); henryshome@gmail.com (P.-C.L.); nice302a@gmail.com (C.-C.L.); jasonsyu0131@gmail.com (F.-C.H.); 0117siou@gmail.com (J.-S.L.); 2 Department of Nursing, Chang Gung University of Science and Technology, Taoyuan 333, Taiwan; 3 Department of General Surgery, Jen Ai Hospital, Taichung 400, Taiwan; huangts1234@gmail.com; 4 School of Traditional Chinese Medicine, College of Medicine, Chang Gung University, Taoyuan 333, Taiwan; 5 Department of Pediatrics, Baylor College of Medicine, Texas Children’s Hospital Cancer Center, Houston, TX 77030, USA; hchiu@bcm.edu (H.-S.C.); sumazin@bcm.edu (P.S.); 6 Department of Medicine, School of Medicine, Chang Gung University, Taoyuan 333, Taiwan; steele0618@gmail.com; 7 Department of Physical Medicine and Rehabilitation, Chang Gung Memorial Hospital, Keelung Branch, Keelung 204, Taiwan; 8 Department of Bioscience and Biotechnology, National Taiwan Ocean University, Keelung 202, Taiwan; panghung.ntou@gmail.com; 9 Department of Ophthalmology, Chang Gung Memorial Hospital, Keelung Branch, Keelung 204, Taiwan; chichinsun@gmail.com; 10 Department of Plastic and Reconstructive Surgery, Chang Gung Memorial Hospital, Linkou Branch, Taoyuan 333, Taiwan; 11 Craniofacial Research Center, Chang Gung University, Taoyuan 333, Taiwan

**Keywords:** negative pressure wound therapy (NPWT), dickkopf-related-protein1 (DKK-1), hair follicle stem cells (HFSCs), epidermal cells, inflammatory

## Abstract

Negative Pressure Wound Therapy (NPWT) is a commonly employed clinical strategy for wound healing, yet its early-stage mechanisms remain poorly understood. To address this knowledge gap and overcome the limitations of human trials, we establish an NPWT C57BL/6JNarl mouse model to investigate the molecular mechanisms involved in NPWT. In this study, we investigate the intricate molecular mechanisms through which NPWT expedites wound healing. Our focus is on NPWT’s modulation of inflammatory immune responses and the concurrent orchestration of multiple signal transduction pathways, resulting in shortened coagulation time and reduced inflammation. Notably, we observe a significant rise in dickkopf-related protein 1 (DKK-1) concentration during NPWT, promoting the differentiation of Hair Follicle Stem Cells (HFSCs) into epidermal cells, expediting wound closure. Under negative pressure, macrophages express and release DKK-1 cytokines, crucial for stimulating HFSC differentiation, as validated in animal experiments and in vitro studies. Our findings illuminate the inflammatory dynamics under NPWT, revealing potential signal transduction pathways. The proposed framework, involving early hemostasis, balanced inflammation, and macrophage-mediated DKK-1 induction, provides a novel perspective on enhancing wound healing during NPWT. Furthermore, these insights lay the groundwork for future pharmacological advancements in managing extensive wounds, opening avenues for targeted therapeutic interventions in wound care.

## 1. Introduction

Healthy skin is crucial for safeguarding the body against various threats, and the process of full-thickness skin wound healing involves key stages such as hemostasis, inflammation, proliferation, and remodeling. Factors like immune cells, cytokines, and signaling molecules play pivotal roles in these stages. Hemostasis, vital for stopping bleeding, engages mechanisms like vasoconstriction, platelet release of PDGF, and the coagulation cascade. In the inflammation stage, FGF-21, PPARs, and RAGE are significant contributors [1,2,3]. Conditions like diabetes can hinder wound healing, necessitating advanced therapeutic strategies. Wound healing may be impaired by various factors, creating a demand for medical interventions that enhance healing and prevent complications in challenging cases [4,5].

While various wound care principles aim to promote wound healing, the main treatment methods involve debridement surgery, wound cleansing, and the application of dressings. Advances in technology have led to the development of new dressings, but their superiority over traditional wet dressings remains inconclusive [6,7,8,9]. NPWT has emerged as a mature clinical procedure for managing complex wounds, showing promising results in expediting wound healing, reducing complications and surgical site infections, lowering hospitalization costs, and enhancing the quality of life for patients [10]. Despite numerous studies investigating the effects of NP on wound healing and the involvement of complex signaling pathways, the underlying molecular mechanisms remain poorly understood. Additionally, due to the constraints of human trials, establishing an animal model becomes crucial to uncovering the molecular intricacies driving the therapeutic effects of NP treatment. In our previous research and consistent with existing literature, we observed that an NP regimen of 125 mmHg contributes to the disassembly of cell junctions, promoting epithelial migration and ultimately leading to rapid wound closure, as demonstrated in our wound healing model [11,12,13,14].

In investigating NPWT, we established a murine model using a stepped 125 mmHg NP regimen. This study aimed to understand the cytokine, chemokine, and growth hormone profiles during the hemostasis, inflammation, proliferation, and remodeling phases, providing insights into NPWT’s early-stage mechanisms. Our results revealed that NPWT-induced macrophages secrete DKK-1, promoting HFSC differentiation into epidermal cells and accelerating early wound healing. This study offers valuable insights for targeted therapeutic strategies to optimize NPWT efficacy and improve patient outcomes.

## 2. Results 

### 2.1. NPWT Mainly Shortens the Four Main Periods of Wound Healing

**Figure 1:**
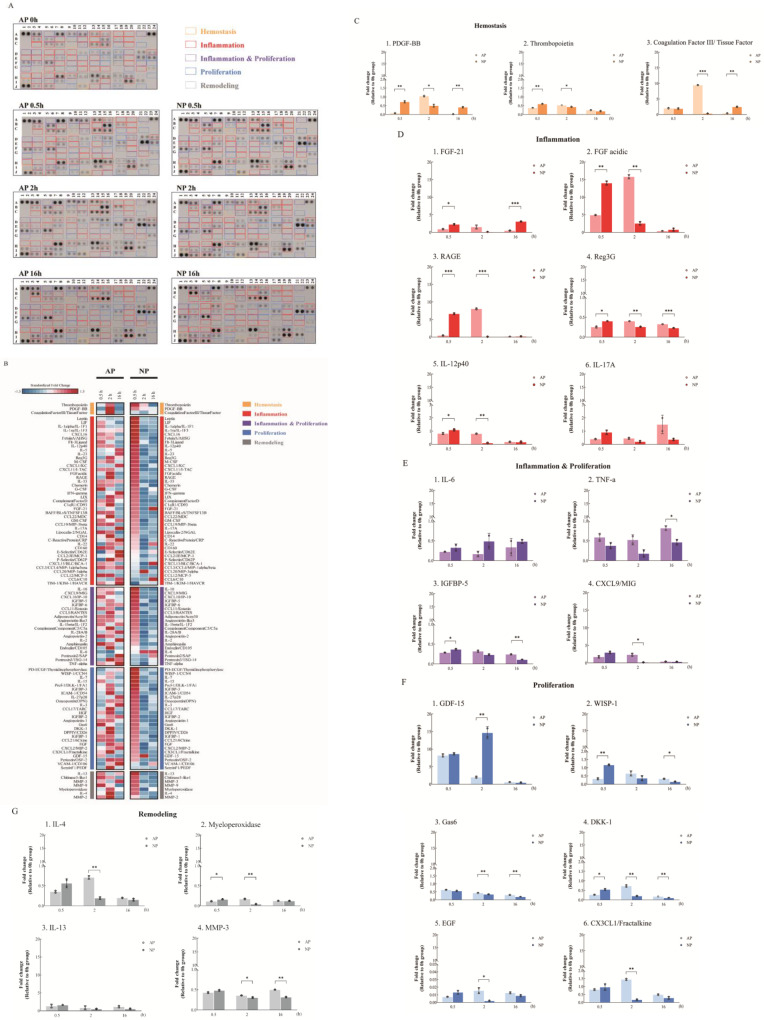
Cytokine array analysis under AP or NP in the early timeline of wound healing. (**A**) Comparison of cytokine expression at 0, 0.5, 2, and 16 h between the AP and NP groups using the cytokine array. The “yellow frames” indicate hemostasis cytokines. The “red frames” indicate inflammatory cytokines. The “purple frames” indicate inflammation and proliferation cytokines. The “blue frames” indicate proliferation cytokines. The “gray frames” indicate remodeling cytokines. (**B**) The heatmap displays standardized expression fold changes, reflecting the extent of these changes in atmospheric (AP) or negative (NP) pressure settings at specific time points compared to the control experiments (0 h). Z−scores for each gene were calculated based on its fold change data from both pressure settings, and genes within the same functional group were sorted based on their Z−scores at the 0.5 h mark in NP. (**C**) Inflammation-related, (**D**) inflammation and proliferation, (**E**) proliferation, (**F**) and remodeling (**G**) cytokine expression. Statistical significance: * *p* < 0.05, ** *p* < 0.01, *** *p* < 0.001 Student’s *t*-test); data shown as mean ± SD.

A cytokine array analysis on serum samples from mice undergoing AP or NP wound therapy at early stages (0, 0.5, 2, and 16 h) is shown in Figure 1A, following the use of the Mouse XL Cytokine Array Kit (R&D). Figure 1B presents a color-coded heatmap for the four stages of full-thickness skin wound healing, aligning with signaling theory, emphasizing the temporal persistence of cytokine expression.

Hemostasis, crucial for stopping bleeding after vascular injury, was analyzed in mice with full-thickness skin wounds treated using AP and NP therapies. The cytokine assay revealed a notable increase in PDGF-BB and thrombopoietin levels at 0.5 h in NP. However, at the 2 h mark, an increase in PDGF-BB and Coagulation Factor III (CFIII) was observed in AP (Figure 1C). In contrast to AP, NP showed a significant decrease in PDGF-BB and CFIII at 2 h, highlighting differences between NP and AP treatment. These findings, along with observations of wound hemostasis, suggest that NPWT promotes blood clot formation by upregulating PDGF-BB, thrombopoietin, and CFIII. Importantly, these hemostatic factors experience an earlier and more pronounced upregulation under NP conditions compared to AP, underscoring the effectiveness of NPWT in promoting hemostasis.

Inflammation-wise, in comparing serum samples from AP- and NP-treated mice, a significant elevation in the expression of FGF-21, FGF acidic, and RAGE was observed at 0.5 h in the NP group, surpassing levels in the AP group. Furthermore, the reduction in expression at 2 h after full-thickness skin wounding was notably faster in the NP group. Interestingly, the AP-treated group exhibited a significant increase in levels of inflammation-related cytokines, including FGF acidic, FGF-21, and RAGE, at 2 h, followed by a reduction at 16 h (Figure 1D). Combining the findings from Figure 1A,B strongly indicates that the NP group experiences an earlier progression through the four stages of wound healing compared to the AP group. Regenerating islet-derived protein 3G (Reg3G) and antimicrobial peptides and proteins (AMPs) were induced 0.5 h after full-thickness skin wounding with NP treatment. AMPs, aside from their antimicrobial functions, also influence keratinocyte (KC) growth, differentiation, cytokine production, and adaptive immunity [15,16,17] (Figure 1D).

The dermal γδ^+^T cells then travel to the draining lymph nodes, where they sustain the activation by triggering dermal dendritic cells to release interleukin IL-12 and generating IL-17 in response to infection. The recruitment of plasmacytoid dendritic cells to the healing skin following injury or infection plays a crucial role in facilitating wound re-epithelialization. In our results, IL-12 significantly increased in the AP-treated group at 2 h and reduced at 16 h. In contrast, IL-12 significantly increased in the NP-treated group at 0.5 h and reduced at 2 h (Figure 1D). Although IL-17 did not reach statistical significance, the trend was consistent.

Due to the experimental design, significant changes related to hemostasis and inflammation can be observed in the early stages of NP wound healing. However, it is challenging to observe comprehensive changes in cytokines associated with proliferation (Figure 1E–G). Interestingly, after achieving hemostasis, the epithelium normalizes the interstitial context by removing fibroblasts stimulated during matrix reconstruction while wound healing is completed. However, Growth-differentiation-factor-15 (GDF-15) acts as a regulator, preventing excessive proliferation and activation of fibroblasts, and induces epidermal changes at 2 h as revealed in our analysis of post-full-thickness skin wounding in the NP groups (Figure 1A,F) during the inflammation stage [18]. This phenomenon did not occur in the AP group.

Of particular interest are the Wnt/β-catenin and Notch signaling pathways, which are involved in hair follicle formation, re-epithelialization, and the generation and deposition of collagen during wound healing. DKK-1 is known to downregulate its expression during the late stages of wound healing, effectively excluding competition with the Wnt/β-catenin pathway [19]. DKK-1 expression was downregulated at 2 h post-full-thickness skin wounding in mice treated with NP (Figure 1A,F).

**Figure 2:**
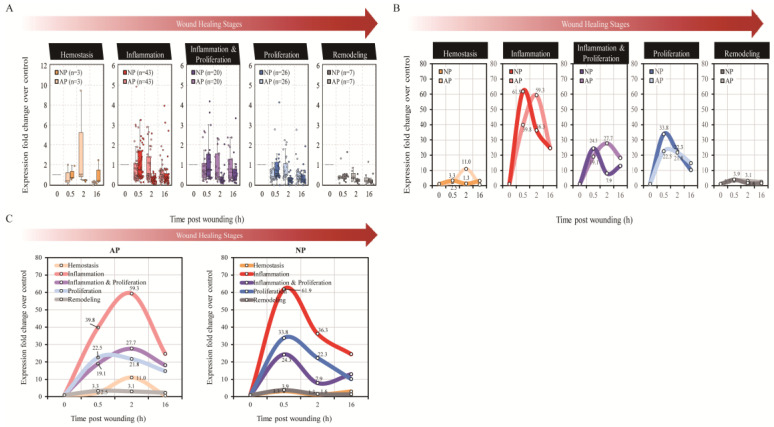
Boxplots facilitate and line plots of cytokine array analysis. (**A**) The boxplots facilitate the comparison of gene-level expression fold changes between the two pressure settings and across multiple time points. The number of genes within each functional group is indicated in parentheses. (**B**) The line plots reveal distinct expression patterns within each functional group, particularly at 0.5, 2, and 16 h, across different pressure settings, and (**C**) similar observations applied to various functional groups with the same pressure settings.

**Figure 3:**
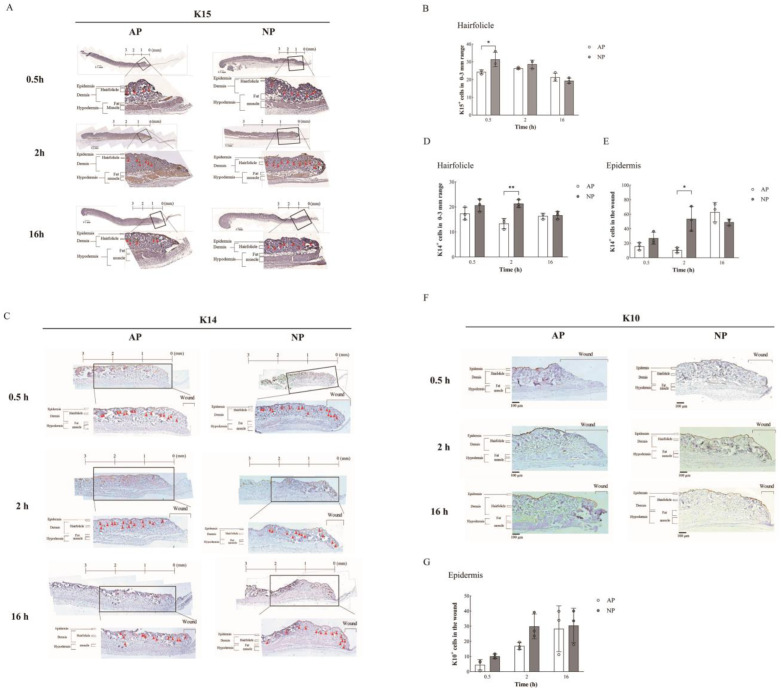
Immunohistochemical analysis of wound healing and hair follicle regeneration. (**A**) K15 expression: Immunohistochemical staining (brown) at 0.5, 2, and 16 h under AP and NP. Insets show magnified regions. Distance from the wound edge is indicated. Arrows indicate hair follicle locations. (**B**) Hair follicle numbers, bulge-derived K15^+^ cells, within 0–3 mm from the wound edge were quantified. (**C**) K14 expression: Immunohistochemical staining (brown) of K14 expression. Insets reveal enlarged regions, with red triangle arrows indicating the location of hair follicles and the distance marked from the wound edge. (**D**) Hair follicle numbers, bulge-derived K15^+^ cells, were quantified. (**E**) The epidermal K14 expression: Statistical analysis of K14 expression in the epidermis of the wound surface (n = 3). (**F**) K10 expression: Immuno-histochemical staining (brown) of K10 expression. (**G**) Insets show magnified regions. K10 expression in the epidermis of the wound surface was quantified. Statistical significance assessed via Student’s *t*-test. Data presented as mean ± SD. * *p* < 0.05, ** *p* < 0.01, compared to mice treated with AP or NP.

Due to our limited observation time of 16 h, we were unable to examine the stages of proliferation and skin remodeling in the full-thickness skin wound healing of mice. Subsequently, we classified the results of the cytokine array into five stages based on known literature: hemostasis, inflammation, inflammation and proliferation, proliferation, and skin remodeling. Following this, we multiplied each raw intensity value by the corresponding normalization factor to obtain standardized intensity values. The expression FC of specific genes under experimental conditions (AP or NP at 0.5, 2, or 16 h) was determined as the ratio relative to the control group (AP at 0 h), using the average of the normalized intensity values across replicates. Each gene profiled on the array was then classified into one of five distinct functional groups: hemostasis, inflammation, inflammation and proliferation, proliferation, and remodeling (Figure 1A). Initially, boxplots were employed to facilitate the comparison of gene-level expression fold changes between the two pressure settings and across multiple time points (Figure 2A). Subsequently, linear line plots derived from these boxplots demonstrated distinct expression patterns within each functional group, particularly at 0.5 and 2 h, across different pressure settings. Similar observations applied to various functional groups under the same pressure settings (Figure 2B). Interestingly, we found that cytokines related to hemostasis, in comparison between NP and AP, continued to be expressed at 2 h in the AP group during wound healing. However, NP had already completed the expression of hemostasis-related cytokines. This result is consistent with the observed phenomenon in experimental animals, suggesting that NP terminates the hemostasis stage earlier compared to AP. Moving on to the second stage of wound healing, inflammation, a notable observation from Figure 2B and Figure 3A, is the pronounced peak in NP at 0.5 h, followed by a rapid decline at 2 h. This indicates that NP contributes to balancing inflammation, facilitating the healing of the wound. In the subsequent stage, inflammation and proliferation, a similar trend is observed, with late-stage inflammation-related cytokines in NP showing a peak at 0.5 h and a rapid decline at 2 h, whereas AP does not exhibit such a trend. The expression of cytokines related to proliferation and skin remodeling is almost negligible. This is attributed to our earlier time limit of 16 h, which is insufficient to observe the proliferation and skin remodeling stages of wound healing.

In summary, NP treatment induces changes in the expression time and number of cytokines, initiating a series of signal transduction pathways that accelerate hemostasis, reduce the immune-inflammatory response, and expedite wound healing. The illustration in Figure 2C summarizes the results of this early wound healing experiment.

### 2.2. Enhanced Wound Healing and Hair Follicle Regeneration through NP Therapy

Recent studies challenge the traditional view of static bulge cells in unwounded skin. In wound healing, hair follicle bulge cells shift dynamically from unipotent to multipotent states through reciprocal signaling with dermal papilla fibroblasts via the Wnt/β-catenin pathway. Interfollicular stem cells generate migrating KCs, emphasizing the dynamic and essential role of hair follicle bulge cells. Post-injury, hair follicle unipotent stem cells transition to a multipotent state guided by dermal papilla fibroblasts through the Wnt/β-catenin/DKK-1 pathway. This signaling not only facilitates epidermal reconstitution but also induces fibroblasts to become myofibroblasts, contributing to wound contraction. Keratin14^+^/β1^-^−integrin-positive stem cells in the basal layer of the epidermis play a pivotal role in the wound healing process. Subsets like CD34^+^/Sox9^+^/keratin15^+^ in the mid-bulge and LRIG1^+^ in the upper bulge maintain skin homeostasis in uninjured skin [20]. These bulge-derived KCs exhibit robust expression of markers such as K15, tumor protein p63, K14, integrin subunit alpha 6, and cytokeratin 19. Therefore, in our study, we employed K15 and K14 as markers for hair follicle bulge and basal-layer epidermal cells, respectively, along with cytokeratin 10 (K10), a marker for spinous-layer epidermal cells. We used IHC to verify the differentiation of cells in the skin tissue surrounding the wound and compared their differentiation patterns under normal and NP conditions [21].

DKK-1 is a secreted protein that functions as a negative regulator of Wnt signaling to prevent hair follicle neogenesis but not HFSC re-epithelialization [22,23]. According to our results, DKK-1 expression was upregulated after 0.5 h, and a rapid reduction in DKK-1 expression was observed at 2 and 16 h after we analyzed the post-full-thickness skin wounds in mice treated with NP (Figure 1A,F). Stem cells are activated and recruited from different skin regions during wound healing. Lineage restriction and spatial confinement of resident skin stem cells were transiently lost during repair, allowing the contribution of multiple epidermal stem cells. HFSCs rapidly migrated from the bulge to the wound and contributed to epidermal repair. Bulge stem cells are in the permanent lowest portion of the hair follicle and provide high plasticity during wound healing [24].

To gain further insights into early wound healing quality, we assessed hair follicle regeneration and re-epithelialization through IHC staining. Specifically, we performed immunohistochemical staining for K15 expression at various time points (0.5, 2, and 16 h) under both AP and NP conditions.

As previously described [20], bulge-derived K15 served as a reliable biomarker for monitoring hair follicle regeneration and re-epithelialization in cutaneous wounds. Notably, we focused our analysis on the region within 0–3 mm around the wound edge, as depicted in Figure 3A. Insets in Figure 3A indicate the magnified regions for visual reference. Figure 3B quantitatively illustrates the presence of hair follicle bulge cells, specifically K15^+^ cells derived from the bulge, in the 0–3 mm region around the wound. Interestingly, at 0.5 h, AP showed a 24.3% presence, while NP exhibited a 31.3% presence in hair follicle bulge cells (K15^+^ cells), indicating a 7% increase under NP compared to AP conditions.

**Figure 4:**
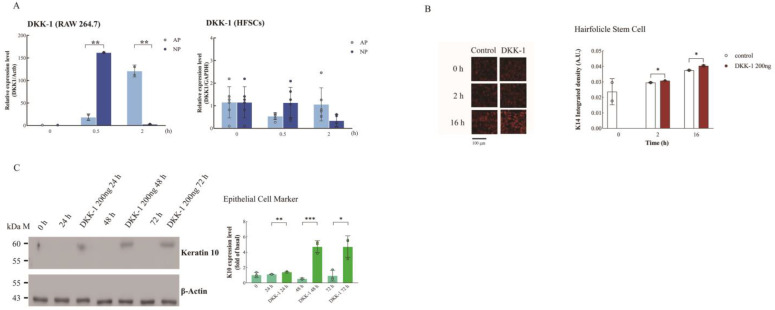
Upregulation of DKK-1 expression by NP in macrophages and its induction of HFSC differentiation. (**A**) RT-qPCR analysis of DKK-1 expression in HFSCs and macrophages under AP or NP conditions at various time points. Note: HFSCs show no expression of DKK-1 under both NP and AP conditions. (**B**) HFSCs treated with 200 ng DKK-1 for 2 h were examined by confocal microscopy, with K14 (red) expression quantified using ImageJ. (**C**) HFSCs treated with 200 ng DKK-1 for indicated intervals (0, 24, 48, and 72 h), and K10 protein levels were determined by Western blotting. Statistical significance: * *p* < 0.05, ** *p* < 0.01, *** *p* < 0.001 (Student’s *t*-test); data presented as mean ± SD.

Additionally, we extended our investigation to include K14, which is another crucial marker for basal-layer epidermal cells, to further elucidate the progression of hair follicle regeneration and re-epithelialization. Similar to K15, we analyzed K14 expression through immunohistochemical staining (Figure 3E) and quantified hair follicle numbers and bulge-derived K15^+^ /K14^+^ cells within the 0–3 mm region from the wound edge (Figure 3D). The conversion from bulge-derived K15 to the basal-layer epidermal cell marker K14 was notably observed around 2 h after initiating NP treatment. Our analysis revealed a significant 8% increase in K14^+^ cells under NP conditions compared to AP at the 2 h mark of treatment. This transition from K15 to K14 expression is a significant event contributing to the accelerated wound healing observed under NP treatment, as it signifies hair follicle re-epithelialization. This process is crucial for wound healing, with Hair Follicle Stem Cells (HFSCs) in the skin bordering the wound playing a pivotal role. Statistical analysis further affirmed the significance of this transition, indicating a pronounced increase in hair follicle re-epithelialization (K15 to K14 conversion) under NP treatment compared to AP treatment, as illustrated in Figure 4B.

This may contribute to accelerated wound healing under NP treatment. Hair follicle re-epithelialization is pivotal in this process, with HFSCs at the wound border significantly contributing. 

Moreover, we observed the difference in the basal-layer epidermal cell markers K14 and K10, which are markers for spinous-layer epidermal cells in the wound epidermal area. We observed a significant increase of approximately 43% in the number of epidermal cells marked by K14 after 2 h of NP treatment. At the same time, we observed here that the expression of K14 in epidermal cells started to increase after a delay of 16 h under normal pressure treatment (From 10.58% to 62.89%, Figure 3E). However, we could not see a difference in K10, a marker for spinous-layer epidermal cells, within 16 h. It is speculated that it may be related to the wound healing time. This part of the study requires longer observations (Figure 3F,G).

Remarkably, the count of epithelial lineages within the hair follicle notably surged following NP treatment, particularly at the 0.5 to 2 h mark (Figure 3B,D). These findings underscore the active phenomenon of hair follicle regeneration and re-epithelialization induced by NP treatment.

### 2.3. DKK-1 Induction Enhances Differentiation of HFSCs into Epithelial Cells

Next, based on the results of the cytokine array analysis of serum at different time points before and after NP treatment (Figure 1), we sought to identify cytokines that might regulate the differentiation of HFSCs into epidermal HFSCs.

Interestingly, recent research has highlighted the role of DKK-1 in promoting the differentiation of HFSCs into epidermal cells, contributing to the restoration of the epidermis and wound closure [4,6]. In line with our findings, we observed that DKK-1 was upregulated earlier under NP treatment, at 0.5 h, compared to the control group, where it was expressed at 2 h (Figure 1F). This indirectly supports our hypothesis that DKK-1 could be one of the factors responsible for accelerated wound healing under NP treatment.

To identify cells expressing and secreting DKK-1 and influencing HFSC differentiation, we cultured HFSCs and macrophages (RAW264.7) in an NP chamber. RT-qPCR confirmed induced DKK-1 expression by macrophages after NP treatment at 0.5 h, not by HFSCs (Figure 4A).

Examining DKK-1’s impact on cell migration, we observed no effect on HFSC migration (Figure S2). HFSC differentiation into KCs is crucial for wound repair. HFSCs express K15, differentiating into basal proliferating-layer cytokeratin 5/14 [25], and further into spinous cells expressing cytokeratin 1/10 [26]. Immunofluorescence showed a significant increase in K14 when HFSCs were treated with DKK-1 from 2 h to 16 h (Figure 4B).

Through Western blotting, K10 expression increased in HFSCs 48–72 h post-DKK-1 treatment (Figure 4C). These results suggest that DKK-1 induces HFSC differentiation into basal and spinous epithelial layers, confirmed by statistical analysis (Figure 4C).

**Figure 5:**
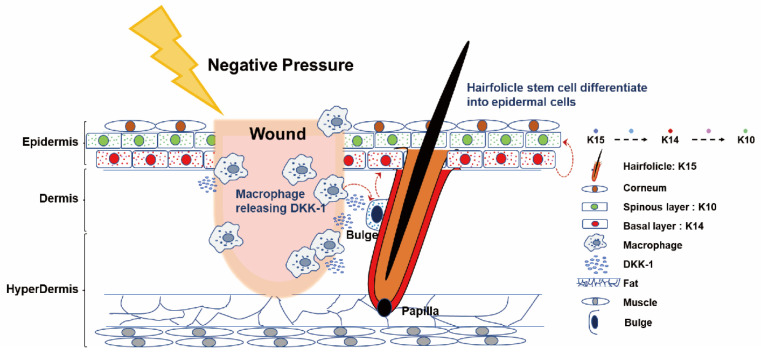
Summary graphic illustration: Cartoon representation of NPWT-induced HFSC differentiation into epidermal cells via macrophage-mediated DKK-1 release in mice. This diagram depicts the NPWT mechanism in mice, where a physical factor induces macrophage-secreted DKK-1 near the wound. DKK-1 stimulates the differentiation of HFSCs from bulge-derived K15^+^ cells into K14^+^ bulge-derived KCs and simultaneously stimulates them to migrate to the vicinity of the wound through an unknown signaling process, differentiating into K10^+^ epidermal cells. NP, acting as a physical factor, simultaneously triggers distinct signaling pathways, regulating hemostasis and immune-inflammatory responses, while accelerating wound healing by promoting HFSC differentiation. This simplification clarifies NPWT’s molecular events, highlighting the interplay between macrophages, DKK-1, and HFSCs. These cells migrate toward the wound, enhancing healing. Skin components and molecular interactions (right) are for reference.

**Figure 6:**
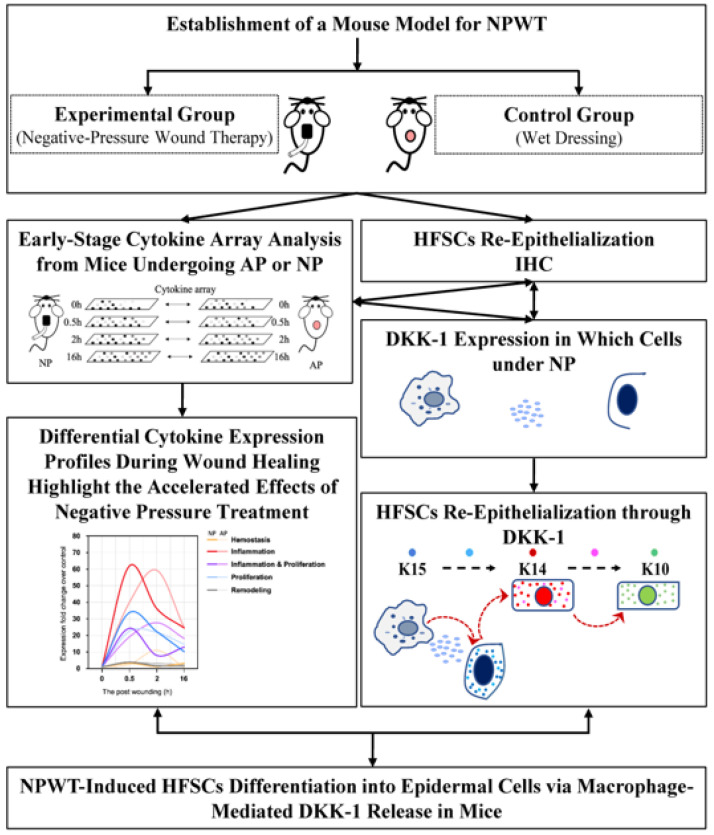
The scheme of this study.

Building on the comprehensive analysis, we propose a compelling hypothesis related to NP treatment. The observed rise in serum DKK-1 levels is suggested to result from macrophage secretion. These DKK-1 molecules act as crucial mediators, initiating signal transduction processes that prompt HFSCs within bulge cells to differentiate into epidermal cells. This cascade of events propels the differentiated cells to actively migrate towards the wound site, significantly accelerating the wound healing process. The hypothesis is visually depicted in Figure 5, enhancing conceptual clarity. Additionally, the experimental design concept of this study is presented in Figure 6. 

## 3. Discussion 

Wound healing is orchestrated by a complex interplay of coagulation factors, paracrine signaling molecules, and immune cells. Pathological conditions like poorly managed diabetes or extensive burns can hinder wound healing efficiency. The clinical success of NPWT in complex wounds is well established, but the intricate molecular mechanisms remain unclear. Due to impracticalities in human trials, establishing an animal model is crucial for unraveling the precise molecular pathways associated with NPWT [27,28,29,30,31,32,33].

This study investigates the molecular mechanisms of NPWT in early wound healing using a mouse model, emphasizing cytokines, chemokines, and growth hormones. The results show dynamic changes in factors related to hemostasis, inflammation, and accelerated wound healing, suggesting NPWT’s efficacy. Despite the 16 h observation limitation, this study provides valuable insights, including a time course chart illustrating early cytokine responses. 

However, it is important to acknowledge certain limitations in our study. The 16 h observation time restricted a comprehensive exploration of the proliferation and skin remodeling stages, leaving opportunities for future investigation. Moreover, while our mouse model successfully simulated NPWT conditions, further in-depth research into the extended signaling transduction regulation and molecular mechanisms of related cytokines reported in our study is warranted. The later stages of wound healing, particularly the proliferation (5–7 days) and remodeling (14–21 days) phases, necessitate additional time points to draw definitive conclusions regarding the facilitative role of NP therapy in these stages [34].

In summary, this research sheds light on the role of macrophage-mediated DKK-1 secretion. Despite acknowledging study limitations, the discussion explores changes in cytokine expression, the phases of accelerated wound healing, and the promotion of HFSCs’ differentiation into epidermal cells. The proposed hypothesis suggests that DKK-1, induced by macrophages under NP conditions, serves as a critical mediator initiating signal transduction processes that contribute to the observed accelerated wound healing. Recognizing the need for extended research into signaling transduction regulation and molecular mechanisms, particularly in the later stages of wound healing, this study provides valuable insights into NPWT’s molecular underpinnings and offers a visual reference for researchers, potentially guiding future investigations.

### Clinical Problem Addressed

Despite the widespread clinical use of NPWT for promoting wound healing, the early-stage molecular mechanisms underlying its effectiveness remain poorly understood. The clinical problem addressed by our research is the lack of comprehensive insights into how NPWT impacts wound healing at the molecular level, especially during the initial stages. Understanding these mechanisms is crucial for optimizing NPWT’s therapeutic potential, ensuring better outcomes for patients undergoing this common wound care modality.

## 4. Materials and Methods

### 4.1. Electronic Laboratory Notebook Platform

The Electronic Laboratory Notebook Platform was not used.

### 4.2. Antibodies and Reagents

Human HFSC frozen vials (Cat #M36007-08), freezing media (Cat # M36007-08FM), serum-free media (Cat #M36007-08), complete media with serum (Cat #M36007-08S), Xeno-free cell dissociation media (Cat #M37001-02CDM), 1× trypsin EDTA (Cat #T1509-014), 1× PBS solution (calcium & magnesium free) (Cat #P1408-013), flasks (Cat #36007-08-T25 and Cat #36007-08-T75), and well plates were obtained from Celprogen (Torrance, CA, USA). DMEM (Dulbecco’s modified Eagle’s medium), PBS (phosphate-buffered saline), FBS (fetal bovine serum), and 1% penicillin/streptomycin were purchased from Gibco (Waltham, MA, USA). DDK1 (NM_012242) human recombinant protein (Cat #TP723065) was purchased from Origene (Rockville, MD, USA). RAW264.7 cells (Cat #TIB-71) were obtained from ATCC (Virginia, MA, USA). Individual chambers (2 block wells, sterile to SAL 10-6 20190) were obtained from SPL^®^ SPLScar™ Block. (Pocheon-si, Republic of Korea). EnVision Detection Systems Peroxidase/DAB [Rabbit/Mouse] (Cat #K5007) was purchased from Dako (Santa Clara, CA, USA). The Proteome Profiler Mouse XL Cytokine Array kit (Cat #ARY028) was purchased from R&D Systems (Minneapolis, MN, USA). DAPI (4’,6-diamidino-2-phenylindole, dihydrochloride) (Cat #D1306) and SuperScript III were purchased from Invitrogen (Carlsbad, CA, USA). TRIzol reagent was purchased from Life Technologies (Carlsbad, CA, USA). BSA (Cat #37520) was purchased from Thermo Fisher Scientific (Waltham, MA, USA). Triton X-100 (Cat #11332481001) and hematoxylin (Cat #H9627) were purchased from Sigma‒Aldrich (St. Louis, MO, USA). The 4% paraformaldehyde (Cat #INOV29) was purchased from BIONOVAS^®^ (Kingsdale Ave, Toronto, Canada). Normal donkey serum (Cat #148087) was purchased from Jackson ImmunoResearch (West Grove, PA, USA). FORANE (isoflurane) was purchased from Aesica (Queenborough, UK). Bain (Nalbuphine) was purchased from Genovate Biotechnology (Hsinchu County, Taiwan). Antibodies employed for immunohistochemistry, immunofluorescence, and Western blotting are detailed in Table S1.

### 4.3. Mouse Care

Eight-week-old male C57BL/6JNarl mice were purchased from the National Laboratory Animal Center (Taipei, Taiwan). The animal experiments were approved by the Institutional Animal Care and Use Committee (IACUC) of Chang Gung Memorial Hospital (IACUC-2016071301).

### 4.4. Establishment of a Mouse Model for NPWT

We implemented a mouse model to investigate the influence of NPWT on wound healing. The experimental protocol involved the following steps: Eight-week-old male C57BL/6JNarl mice were randomly allocated into two groups—a traditional wet dressing group under atmospheric pressure (AP) and an NP group. At each time point, 2–3 mice were utilized, with data collected at 0, 0.5, 2, and 16 h.

To maintain consistent wound pressure, mice were anesthetized with isoflurane and positioned using a stabilizer, with tranquilizers and analgesics administered as needed. Subsequently, the dorsal hair of the mice was removed using electric shavers, followed by complete depilation using depilatory cream. A 6 mm diameter full-thickness wound was then created in the dorsal region of each mouse utilizing a biopsy punch (Figure S1A). To preserve the wound’s shape, a 0.2 cm thick artificial skin layer was applied around the wound edge (Figure S1B). The wound was covered with a dressing, and NP therapy was administered using the Apex NP therapy device at a pressure of 125 mmHg for varying treatment durations. Mice were sacrificed at different time points, and serum, along with tissue surrounding the wound, was collected for analysis.

This mouse model effectively simulated skin injury and replicated the NPWT procedure within a controlled environment. The experimental design allowed for an investigation into the impact of NP therapy on wound healing while controlling for potential confounding factors. By employing different treatment durations under a consistent NP regimen, we were able to discern time-dependent responses to NPWT.

In the subsequent analysis, our focus centered on evaluating the effects of NPWT on wound healing across four distinct stages. 

### 4.5. NPWT

To assess the impact of NPWT on wound recovery in mice, we conducted a randomized division of mice into two groups: one subjected to traditional wet dressing under AP and the other to NP wound therapy. Each experimental group comprised 2–3 mice for analysis of outcomes. Mice were anesthetized with isoflurane (4–5% for induction and 3% for maintenance anesthesia). The mice were fixed with a stabilizer during this procedure and given appropriate tranquilizers (diazepam: 5 mg/kg intraperitoneally) and analgesics (ketorolac: 0.7–10 mg/kg, 24 h orally) to ensure that the wound pressure remained stable. Then, the hair on the back of the mice was removed using electric shavers, and depilatory cream was used for complete removal. A biopsy punch (Kai Industries, Co., Ltd., Seki-shi, Japan) was used to produce a 6 mm diameter full-thickness wound on the backs of the mice. Next, a 0.2 cm thick layer of artificial skin was applied to the edge of the wound to fix the shape of the wound. After the wound was covered with a dressing, an NP therapy device manufactured by Apex (ZIP-S, Apex Medical Corp., Pingtung City, Taiwan) was used to treat the wound with an NP of 125 mmHg for different treatment times.

### 4.6. Immunohistochemical (IHC) Staining and Hair Follicle Count

After the NPWT experimental steps, mouse skin wound tissue (including new tissue and original tissue) was measured to determine the size of the epidermal tissue along the edge of the wound for paraffin embedding to perform IHC staining and hair follicle counts. Mice were euthanized via carbon dioxide inhalation. To examine the expression and location of cytokeratin 15 (K15), IHC staining was performed on sections of AP and NP wound skin tissues at different time points. Sections were deparaffinized and rehydrated. Antigen retrieval was performed using a citric acid buffer at 95 °C for 20 min, followed by washing three times with PBS. Endogenous peroxidase activity was quenched using 3% H_2_O_2_ in methanol at room temperature for 20 min. Nonspecific binding was blocked by preincubation with PBS containing 5% BSA for 30 min at room temperature. The sections were incubated with anti-K15 at 4° C overnight, followed by an anti-mouse antibody at room temperature for 1 h. Bound antibodies were detected using the DAB substrate (1 mL substrate buffer mixed with 20 μL DAB chromogen). Finally, the tissue was counterstained with hematoxylin for 1 min and mounted. The slides were observed under a microscope, and the hair follicle bulge cells, specifically bulge-derived K15^+^ cells, were counted using TissueFAXS Viewer 5.0 (Vienna, Austria). Antibodies employed for immunohistochemistry are detailed in Table S1.

### 4.7. Cytokine Array Assay

We collected 500 µL of blood in 1.5 mL Eppendorf tubes from the cheeks of the mice after AP or NP treatment before euthanasia. After setting for 0.5–2 h, centrifugation was performed at 1500× *g* and 4 °C for 10 min. The supernatant was transferred into a new tube and centrifuged at 13,000× *g* and at 4 °C for 3 min to obtain the serum. Serum cytokine levels were analyzed using the mouse XL cytokine array (R&D Systems, USA) according to the manufacturer’s instructions. Serum samples (20 µL) from the AP or NP groups were combined for each test for a final volume of 100 µL for each array membrane. The arrays were analyzed using the UVP imaging system and exposed for 10 min, with one picture recorded every 3 min. The results were quantified using ImageJ software (JAVA 1.8.0, USA). 

### 4.8. Cytokine Array Analysis

The raw intensity values from the cytokine array were normalized using a widely recognized global adjustment method [35]. Specifically, for each array, we calculated a replicate-specific normalization factor ($F_{i}$) based on the mean intensity across three positive references (${\bar{R}}_{i}$), each measured in duplicate spots, using the formula $F_{i}=min(1,\;{\bar{R}}_{(i\%2)+1}/{\bar{R}}_{i})$. Here, i represents the index of the replicate, which is either 1 or 2, min refers to taking the minimum between values, and % represents the modulo operation. Subsequently, we multiplied each raw intensity value by the normalization factor of the corresponding replicate to obtain the normalized intensity value. The expression fold change (FC) of a specific gene under an experimental condition (AP or NP at 0.5, 2, or 16 h) was determined as the ratio relative to the control group (AP at 0 h) using the average of the normalized intensity values across replicates. In addition, each gene profiled on the array was classified into one of five distinct functional groups: hemostasis (3 genes), inflammation (43 genes), inflammation and proliferation (20 genes), proliferation (26 genes), and remodeling (7 genes). When calculating the FC for each functional group, we computed the mean FC across all gene members within the same group. The raw data were submitted to GEO (GSE252915).

### 4.9. Culture of Human Hair Follicle Stem Cells (HFSCs)

In this study, Human Hair Follicle Stem Cells (HFSCs) were utilized, obtained from Celprogen under catalog number 36007-08. The cells were provided in a frozen ampule, containing 1.2 × 10^6^ cells, sourced from the Human Frontal region Scalp’s Hair Follicle bulge. For storage, the frozen vial was maintained in the liquid nitrogen vapor phase. Thawing procedures were crucial for cell viability. The cryovial, retrieved from dry ice or liquid nitrogen vapor phase, was swiftly transferred to a 37 °C water bath or 37 °C dry oven in a shaker for less than 1–2 min. Subsequently, the thawed cells were promptly diluted in complete growth media with serum to minimize potential toxic effects. Prior to experimentation, thorough assessments were conducted. Mycoplasma tests, employing both PCR and agar methods, yielded negative results. Sterility checks confirmed the absence of bacteria, yeast, and mold. Donors underwent pre-screening, testing negative for infectious diseases such as ABO/RH, Hepatitis B Surface Antigen, HIV1 and 2, Syphilis, and others. The HFSCs expressed positive markers, including bl-integrin, Keratin 15, Keratin 19, CD71, SA 200, S100, CK19, CK14 (10%), CD34, CD133, Nestin, Ki67, Sox 2, Neurogenin 3, Map 2, O 2A, S-100B, and NF. 

Cell culture conditions were meticulously controlled. Maintained at 37 °C in a 5% CO_2_ humidified incubator, the cells exhibited a mixed population with approximately 95% attached cells and 5% in suspension. Daily media changes were imperative after 48 h or upon observing a change in color to a slight yellow from pink. The culture was characterized by a rapid growth rate, with a population doubling time of 24 h. For sub-culturing, thawed cells were transferred to a sterile centrifuge tube, washed, and centrifuged at 100× *g* for 7 min. The resulting cell pellet was resuspended in Human Hair Follicle Stem Cells Complete Growth Medium. Subsequently, the cells were added to pre-coated flasks with Human Hair Follicle Stem Cells Extracellular Matrix, incubated at 37 °C in a 5% CO_2_ humidified incubator. Media changes were performed every 24 to 48 h, with a sub-culturing ratio of 1:3 depending on cell density. Seeding cells from plated tissue culture flasks involved wiping with 70% ethanol, washing with 1× PBS sterile solution, trypsinization, neutralization with Human Hair Follicle Stem Cells Culture Complete Growth Media with Serum, and centrifugation at 100× *g* for 7 min. The cells were then plated (5 × 10^5^) on pre-coated flasks with Human Hair Follicle Stem Cells Culture Extracellular Matrix. In addition to the HFSCs, specific reagents were utilized, such as Freezing Medium (Celprogen, Cat# M36007-08FM), and Trypsin (Celprogen, Cat# T1509-014). PBS at 1× was used as a sterile solution (Celprogen, Cat# P1408-013). 

Comprehensive details, protocols, flow diagrams, and videos can be found at https://celprogen.com/human-hair-follicle-stem-cells-frozen-vial/. Note: All procedures strictly adhered to the guidelines provided by Celprogen and followed the specific instructions outlined in Section 4.

### 4.10. Cell Migration Analysis

HFSCs were cultured in a chamber or block and then clamped to the insert after the cells grew to 80% confluence. The cells were placed in serum-free medium for 16 h and treated with or without DKK-1 (200 ng) for 0, 2, and 16 h. At each time point, the medium was removed, the cells were washed with PBS, and they were observed using a microscope and ImageJ software (JAVA 1.8.0, USA).

### 4.11. Immunofluorescence Analysis

The cells were fixed in 4% formaldehyde for 20 min, permeabilized with 0.1% Triton X-100-PBS, and blocked in 10% normal donkey serum (Jackson ImmunoResearch). Antigen localization was performed by incubation with the appropriate antibodies at 4 °C overnight. The secondary antibodies conjugated to Alexa Fluor 488, Alexa Fluor 568, or Alexa Fluor 647 (Molecular Probes) were then applied for another 2 h incubation at 25 °C. DNA staining was performed using DAPI (4,6-diamidino-2-phenylindole) (Molecular Probes, Eugene, OR, USA). The images were observed under a confocal microscope (Leica TCSSP2; Leica Microsystems Inc., Buffalo Grove, IL, USA).

### 4.12. Western Blot

Western blot procedures were performed as described in ref [36]. Antibodies employed for Western blot are detailed in Table S1.

### 4.13. RT-qPCR

Total RNA from HFSCs or RAW264.7 cells was extracted using a commercial TRIzol reagent. RNA (5 μg) was reverse-transcribed in a final volume of 20 μL using SuperScript III (Invitrogen) following the manufacturer’s protocol. qPCR amplification was conducted using Rotor-Gene Q (Corbett-Research, Mortlake, Australia). PCR products were monitored by measuring the fluorescence of the intercalated SYBR Green (Qiagen, Hilden, Germany). The primers used were mDKK-1 (F:5’-TCTCTATGAGGGCGGCAACA-3’ and R:5’-TTTCGGCAAGCCAGACAG AT-3’) and hDKK-1 (F:5’-GAGTACTGCGCTAGTCCCAC-3’ and R:5’-TGGAATACCCATC CAAGGTGC-3’), respectively. The expression of DKK-1 was normalized to that of commercial Mm_Actb_2_SG (ID: Actb: QT01136772) and commercial Hs_GAPDH_2_SG (ID: Gapdh: QT01192646), respectively.

### 4.14. Statistical Analysis

Statistical analysis was performed using GraphPad Prism 8.0 software (GraphPad, San Diego, CA, USA). Student’s *t*-tests were conducted with a significance level set at *p* ≤ 0.05. The data are presented as mean ± standard error of the mean (s.e.m.) or standard deviation (SD), as specified in the Supplemental Excel. Cytokine production quantification from the cytokine arrays was based on the average of duplicate values from two independent experiments, with statistical significance determined using a *t*-test. Data are expressed as mean ± SD, with significance denoted as * *p* < 0.05 and ** *p* < 0.01. Our choice of the Student’s *t*-test for analyzing expression fold changes between pressure settings (AP and NP) was based on its suitability for scenarios with large effect sizes and small sample sizes, as supported by De Winter 2013. Simulations from this study confirmed the efficacy of Student’s *t*-test in controlling Type I errors at 5% for sample sizes as small as 2–5 when effect sizes exceed 80%. Our data align with these criteria, showing substantial effect sizes across time points measured by Cohen’s d (370% for 0.5 h, 797% for 2 h, and 476% for 16 h). Supplementary Figure S3 provides a boxplot illustrating the distribution of effect sizes across all tested genes, further justifying our choice of Student’s *t*-test. References to experimental and analytical methods can be found in [37,38].

## Data Availability

All data are contained within the article and supplementary materials.

## Supplementary Materials

The following supporting information can be downloaded at https://www.mdpi.com/article/10.3390/ijms25042373/s1.

## Abbreviations

Antimicrobial peptides and proteins (AMPs); Atmospheric pressure (AP); Antimicrobial peptides and proteins (AMPs); Cytokeratin 10 (K10); Cytokeratin 15 (K15); Dickkopf-related protein 1 (DKK-1); Fold change (FC); Growth-differentiation-factor-15 (GDF-15); Hair Follicle Stem Cells (HFSCs); Immunohistochemical (IHC); Institutional Animal Care and Use Committee (IACUC); Keratinocytes (KCs); Negative pressure (NP); Negative Pressure Wound Therapy (NPWT); Regenerating islet-derived protein 3G (Reg3G)
