# Supplementary material for: Deciphering Early-Stage Molecular Mechanisms of Negative Pressure Wound Therapy in a Murine Model
Source: Int J Mol Sci. 2024 Feb 17;25(4):2373. doi: 10.3390/ijms25042373 (PMC10888958; doi:10.3390/ijms25042373)
Supplement: Supplementary file 1 [file ijms-25-02373-s001.zip › ijms-2862185-supplementary.pdf]

Supplementary Materials

Supplementary Figure and Figure Legends

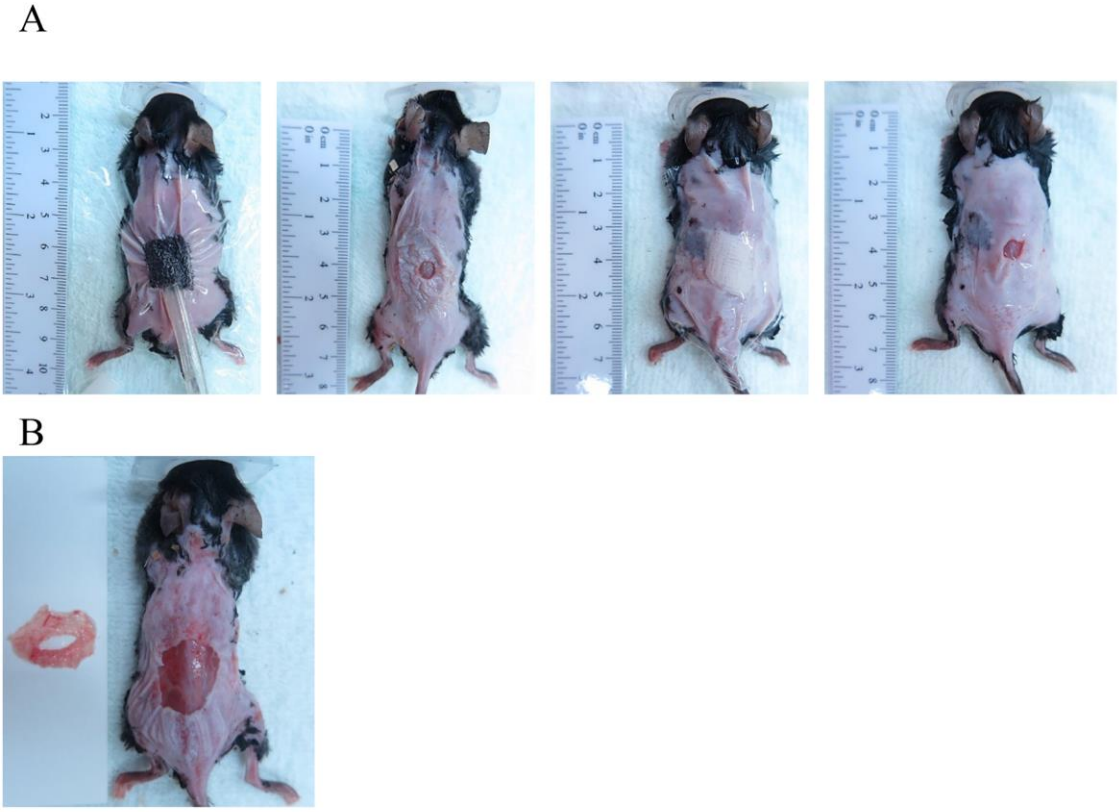

Figure S1 Mouse dorsal wound site for NPWT and AP.

(A) The left images depict the dorsal wound site conditions during NPWT, while the right images illustrate the conditions during AP treatment. (B) These images display the skin that was removed from around the wound site for reference.

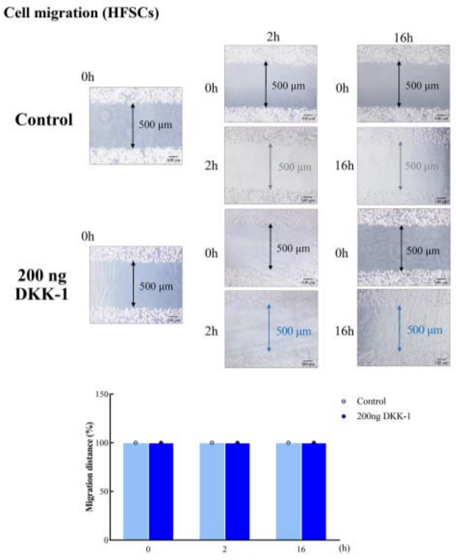

Figure S2 Migration Analysis of HFSCs.

HFSCs were treated with 200 ng DKK-1, and their migration was monitored over time using ImageJ.

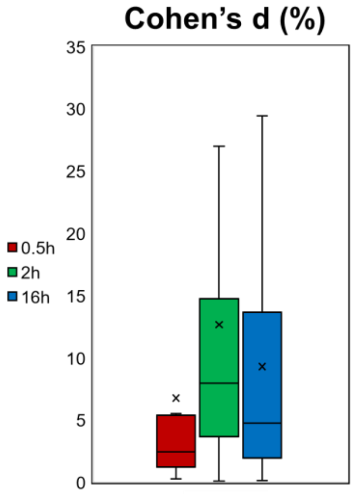

Figure S3 The box plot illustrates the distribution of effect sizes across all tested genes, with red representing 0.5h, green representing 2h, and blue representing 16h.

Supplementary Table

Table S1 Antibodies used for immunohistochemistry, immunofluorescence and western blot.

Antigen	Manufacturer	Clone	Isotype	Dilution
Anti- Cytikeratin 10 antibody (Cat #ab76318)	Abcam, Cambridge, MA, USA	Rabbit monoclonal [EP1607IHCY]	IgG	WB:1:250<br>IF: 1:100
Anti-Cytokeratin 14 antibody (Cat #MA5-11599)	Invitrogen, Waltham, MA USA	Mouse monoclonal [LL002]	IgG3	IHC-P: 1:100<br>IF: 1:100
anti-Cytokeratin 15 antibody (Cat #ab80522)	Abcam, Cambridge, MA, USA	Mouse monoclonal [LHK15]	IgG2a	IHC-P: 1:100<br>IF: 1:100
Anti-β-Actin antibody (Cat #A5441)	Sigma-Aldrich, St. Louis, MO,USA	Mouse monoclonal [AC-15]	IgG1	WB:1:20,000
Anti-Mouse IgG-Peroxidase antibody produced in rabbit (Cat #A9044)	Sigma-Aldrich, St. Louis, MO,USA	Polyclonal	IgG	Secondary antibodies<br>WB for Actin: 1: 10,000
Goat anti-Rabbit IgG (Cat #12-348)	Sigma-Aldrich, St. Louis, MO,USA	Polyclonal	IgG	Secondary antibodies<br>WB for K-10: 1:1,000
Alexa Flour 647 donkey anti-mouse (Cat #A31571)	Invitrogen, Carlsbad, CA, USA	Polyclonal	IgG	Secondary antibodies<br>IF for K-14 and K-15: 1:1,000
Alexa Flour 488 donkey anti-rabbit (Cat #A21206)	Invitrogen, Carlsbad, CA, USA	Polyclonal	IgG	Secondary antibodies<br>IF for K-10: 1:1,000

Table S2 Cytokine array list

Coordinate	Analyte/Control	Entrez Gene ID	Alternate Nomenclature
A1, A2	Reference Spots	N/A	
A3, A4	Adiponectin/Acrp30	11450	AdipoQ
A5, A6	Amphiregulin	11839	AR, SDGF
A7, A8	Angiopoietin-1	11600	Ang-1, Angpt1
A9, A10	Angiopoietin-2	11601	Ang-2, Angpt2
A11, A12	Angiopoietin-like 3	30924	ANGPT-L3
A13, A14	BAFF/BLyS/TNFSF13B	24099	CD257, TALL1, THANK, ZTNF4
A15, A16	C1q R1/CD93	17064	AA4 Antigen, C1q Rp, CD93
A17, A18	CCL2/JE/MCP-1	20296	MCAF
A19, A20	CCL3/CCL4/MIP-1α/β	20302/20303	
A21, A22	CCL5/RANTES	20304	SISd
A23, A24	Reference Spots	N/A	
B3, B4	CCL6/C10	20305	MRP-1

B5, B6	CCL11/Eotaxin	20292	
B7, B8	CCL12/MCP-5	20293	
B9, B10	CCL17/TARC	20295	
B11, B12	CCL19/MIP-3β	24047	ELC
B13, B14	CCL20/MIP-3α	20297	exodus-1, LARC
B15, B16	CCL21/6Ckine	18829	exodus-2, SCYA21, SLC, TCA-4
B17, B18	CCL22/MDC	20299	ABCD1, MDC, STCP-1
B19, B20	CD14	12475	
B21, B22	CD40/TNFRSF5	21939	
C3, C4	CD160	54215	Natural killer cell receptor BY55, NK1; NK28
C5, C6	Chemerin	71660	RARRES2, TIG-2
C7, C8	Chitinase 3-like 1	12654	CHI3L1, Cgp39, YKL40
C9, C10	Coagulation Factor III/Tissue Factor	14066	TF, CD142, Thromboplastin
C13, C14	Complement Factor D	11537	Adipsin, C3 convertase activator, Properdin factor D
C15, C16	C-Reactive Protein/CRP	12944	
C17, C18	CX3CL1/Fractalkine	20312	FKN, Neurotactin
C19, C20	CXCL1/KC	14825	CINC-1; GROα; KC; MGSA-α
C21, C22	CXCL2/MIP-2	20310	GROβ, GRO2, CINC-3
D1, D2	CXCL9/MIG	17329	CRG-10, CMK
D3, D4	CXCL10/IP-10	15945	CRG2, C7
D5, D6	CXCL11/I-TAC	56066	H174, SCYB9B
D7, D8	CXCL13/BLC/BCA-1	55985	
D9, D10	CXCL16	66102	SRPSOX
D11, D12	Cystatin C	13010	ARMD11, CST3, γ-trace
D13, D14	DKK-1	13380	Dickkopf-1
D15, D16	DPPIV/CD26	13482	Dpp4, Dipeptidyl-peptidase IV
D17, D18	EGF	13645	Epidermal Growth Factor
D19, D20	Endoglin/CD105	13805	ENG
D21, D22	Endostatin	12822	Col18a1
D23, D24	Fetuin A/AHSG	11625	AHSG, alpha-2-HS-glycoprotein
E1, E2	FGF acidic	14164	FGF-1
E3, E4	FGF-21	56636	
E5, E6	Flt-3 Ligand	14256	Flt3lg
E7, E8	Gas 6	14456	Growth Arrest Specific
E9, E10	GCSF	12985	Csf3
E11, E12	GDF-15	23886	MIC-1
E13, E14	GM-CSF	12981	Csf2
E15, E16	HGF	15234	Scatter Factor, SF, Hepatopoietin-A
E17, E18	ICAM-1/CD54	15894	
E19, E20	IFN-γ	15978	IFNG

E21, E22	IGFBP-1	16006	
E23, E24	IGFBP-2	16008	
F1, F2	IGFBP-3	16009	
F3, F4	IGFBP-5	16011	
F5, F6	IGFBP-6	16012	
F7, F8	IL-1α/IL-1F1	16175	
F9, F10	IL-1β/IL-1F2	16176	
F11, F12	IL-1ra/IL-1F3	16181	IL1RN
F13, F14	IL-2	16183	
F15, F16	IL-3	16187	
F17, F18	IL-4	16189	B cell-stimulatory factor-1
F19, F20	IL-5	16191	
F21, F22	IL-6	16193	
F23, F24	IL-7	16196	
G1, G2	IL-10	16153	CSIF
G3, G4	IL-11	16156	
G5, G6	IL-12 p40	16160	
G7, G8	IL-13	16163	
G9, G10	IL-15	16168	
G11, G12	IL-17A	16171	
G13, G14	IL-22	50929	IL-TIF
G15, G16	IL-23	83430	
G17, G18	IL-27 p28	246779	
G19, G20	IL-28A/B	330496/338374	
G21, G22	IL-33	77125	NF HEV, DVS 27
G23, G24	LDL R	16835	low density lipoprotein receptor
H1, H2	Leptin	16846	OB
H3, H4	LIF	16878	
H5, H6	Lipocalin-2/NGAL	16819	Siderocalin, 24p3
H7, H8	LIX	20311	CXCL5, GCP-2, ENA-78
H9, H10	M-CSF	12977	CSF-1
H11, H12	MMP-2	17390	Gelatinase A
H13, H14	MMP-3	17392	Stromelysin-1
H15, H16	MMP-9	17395	Clg4b, Gelatinase B, GELB
H17, H18	Myeloperoxidase	17523	MPO
H19, H20	Osteopontin (OPN)	20750	Eta-1, Spp1
H21, H22	Osteoprotegerin/TNFRSF11B	18383	OPG, Ocif
H23, H24	PD-ECGF/Thymidine phosphorylase	72962	dThdPase, ECGF1, Gliostatin, MEDPS1, MNGIE
I1, I2	PDGF-BB	18591	
I3, I4	Pentraxin 2/SAP	20219	PTX2
I5, I6	Pentraxin 3/TSG-14	19288	PTX3

I7, I8	Periostin/OSF-2	50706	Fasciclin I-like, POSTN, TRIF52
I9, I10	Pref-1/DLK-1/FA1	13386	DLK1, pG2, ZOG
I11, I12	Proliferin	18811	MRP
I13, I14	Proprotein Convertase 9/PCSK9	100102	NARC-1
I15, I16	RAGE	11596	AGER
I17, I18	RBP4	19662	Retinol-Binding Protein 4
I19, I20	Reg3G	19695	PAP3
I21, I22	Resistin	57264	ADSF, FIZZ3
J1, J2	Reference Spots	N/A	
J3, J4	E-Selectin/CD62E	20339	ELAM1, LECAM2, Sele
J5, J6	P-Selectin/CD62P	20344	GMP-140, LECAM3, Selep
J7, J8	Serpin E1/PAI-1	18787	Nexin, PLANH1
J9, J10	Serpin F1/PEDF	20317	EPC-1
J11, J12	mrombopoietin	21832	Tpo, MGDF
J13, J14	TIM-1/KIM-1/HAVCR	171283	
J15, J16	TNF-α	21926	TNFSF1A
J17, J18	VCAM-1/CD106	22329	
J19, J20	VEGF	22339	VEGF-A, VPF
J21, J22	WISP-1/CCN4	22402	
J23, J24	Negative Control	N/A	
